# ICP-MS trace element analysis in serum and whole blood

**DOI:** 10.1371/journal.pone.0233357

**Published:** 2020-05-20

**Authors:** Nico Laur, Ralf Kinscherf, Karolina Pomytkin, Lars Kaiser, Otto Knes, Hans-Peter Deigner

**Affiliations:** 1 Furtwangen University, Institute of Precision Medicine, VS-Schwenningen, Germany; 2 Department of Anatomy and Cellbiology, University of Marburg, Marburg, Germany; 3 Swiss Analysis AG, Tägerwilen, Switzerland; 4 Institute of Pharmaceutical Sciences, University of Freiburg, Freiburg i. Br, Germany; 5 Fraunhofer Institute IZI, Leipzig, EXIM Department, Schillingallee, Rostock, Germany; 6 Tuebingen University, Faculty of Science, Tübingen, Germany; Queen's University Belfast, UNITED KINGDOM

## Abstract

Trace elements and minerals are compounds that are essential for the support of a variety of biological functions and play an important role in the formation of and the defense against oxidative stress. Here we describe a technique, allowing sequential detection of the trace elements (K, Zn, Se, Cu, Mn, Fe, Mg) in serum and whole blood by an ICP-MS method using single work-up, which is a simple, quick and robust method for the sequential measurement and quantification of the trace elements Sodium (Na), Potassium (K), Calcium (Ca), Zinc (Zn), Selenium (Se), Copper (Cu), Iron (Fe), Manganese (Mn) and Magnesium (Mg) in whole blood as well as Copper (Cu), Selenium (Se), Zinc (Zn), Iron (Fe), Magnesium (Mg), Manganese (Mn), Chromium (Cr), Nickel (Ni), Gold (Au) and Lithium (Li) in human serum. For analysis, only 100 μl of serum or whole blood is sufficient, which make this method suitable for detecting trace element deficiency or excess in newborns and infants. All samples were processed and analyzed by ICP-MS (Agilent Technologies). The accuracy, precision, linearity and the limit of quantification (LOQ), Limit of Blank (LOB) and the limit of detection (LOD) of the method were assessed. Recovery rates were between 80–130% for most of the analyzed elements; repeatabilities (C_v_ %) calculated were below 15% for most of the measured elements. The validity of the proposed methodology was assessed by analyzing a certified human serum and whole blood material with known concentrations for all elements; the method described is ready for routine use in biomonitoring studies.

## Introduction

During the last years, interest in the role of trace elements in biological systems has increased. Almost every metabolic process operates by involving trace elements, ultra-trace elements, or minerals, making them essential for human metabolism; biochemical- and metabolic interactions, very relevant to human medicine as well as in diagnostics and therapy, have already been described in the context of the role of these elements. Especially for trace elements such as Se, Fe, Zn and Cu, but also for minerals such as Na, K, Ca and Mg and other, specific, binding- and transport-proteins could be identified in different membranes of cells, intracellularly and in body fluids [1, 2].

Blood is a body fluid that can be obtained from patients and analyzed to assess the current physiological state of the body. Consequently, serum samples have long been used for identification of disease-related biomarkers and for clinical diagnosis. Whole blood and serum samples have become established for trace element analysis. Elements to be measured were chosen based on their relevance to biological processes.

Sodium (Na), for example, plays a key role in a variety of nerve and muscle functions. Furthermore, potassium (K) and calcium (Ca) are essential for cellular signaling, present in a variety of biological processes, as both are involved in ion channels and pumps [3]. An overload of potassium, called hyperkalemia, for example, can lead to dangerous and possibly deadly changes in heart rhythm, which can also be caused by an overload of calcium. Lithium (Li), in turn, has recently been associated with oxidative stress and high concentrations of lithium obviously exert a positive effect on the extent of it. [4] Magnesium (Mg) is found in a variety of enzymes and cells, such as muscle cells. [5] Also, high levels of manganese (Mn) can lead to toxicity and a neurological disease, similar to Parkinson, called Manganism, making evaluation of actual blood levels beneficial for diagnosis and treatment [6]. Furthermore, haemodialysis patients can experience accumulation of chromium (Cr) and nickel (Ni), but may become deficient in values of manganese, making assessment of blood concentrations essential for supplementation or clearance of corresponding elements. Ni, in turn, facilitates iron (Fe) absorption and may play an important role in the production of red blood cells [7]. The toxicity of Ni excess is, however, associated with cardiovascular, renal and respiratory problems [8]. In addition, chromium (Cr) is important in the metabolism of fats and carbohydrates, as it stimulates fatty acid and cholesterol biosynthesis [9]. Studies like Garbuz et al. [10] demonstrated, that the concentration of Cr can also be used as a monitoring parameter during clinical trials of metal-on-metal hip replacements, as the hip replacements contain Cr and therefore the level of metal ion release may be due to the hip replacements. Zinc (Zn), in turn, is also found in enzymes, catalyzing many synthetic biological reactions [11, 12]. A Zn deficiency is characterized by acral dermatitis, alopecia and diarrhea [13]. Also, iron (Fe) is a trace element with an essential role in oxygen transportation e.g. in hemoglobin of erythrocytes, an Fe deficiency ends in anemia [14]. On the other hand, an Fe overload causes severe consequences in the human body, including liver damage, fibrosis, heart failure, or even cancer [13]. Fe, as well as, Mn are further involved in numerous redox reactions [14,15]. Signs of Mn deficiency include impaired growth, impaired reproductive function, skeletal abnormalities, impaired glucose tolerance and altered carbohydrate and lipid metabolism. Nevertheless, Mn deficiency is not common. It is more of concern for toxicity related to Mn overexposure, which can cause an inflammatory response in the lung, resulting in impaired lung function [16]. In addition, two of the most important trace elements, playing a major role in the development of oxidative stress, are selenium (Se) and copper (Cu). They are mandatory to numerous oxidase and superoxide dismutase (SOD) reactions in the cytosol, especially Cu is a critical functional component of a number of enzymes related to oxidative stress e.g. SOD, cytochrome C oxidase (CCO) in the mitochondria and tyrosinase. [13, 17] As well as Fe and Mn, an excess amount of Cu, is toxic for the human body. An unregulated Cu homeostasis may lead to diseases like Menkes disease, characterized by a deficiency of Cu, or to a disease called Wilson, characterized by excessive overload of Cu [13]. Se, in turn, is an essential micronutrient crucial for many biological functions including the regulation of cell death signaling, in endogenous metabolism and redox reactions [2]. However, an excessive Se overload may lead to selenosis, which is characterized in nausea, chronic fatigue, myasthenia and diarrhea [18]. Indeed, Gold (Au) so far has hardly appeared as an environmental toxin. It is known, however, that some patients have contact allergy due to wearing gold spruce. Furthermore, in human medicine Au salts have been used for the treatment of rheumatoid arthritis since about 60 years [19]. Au has recently also been added to many cosmetic products. An overload with this element causes rashes, gastrointestinal bleeding, nausea, vomiting or even bone marrow damages.

Unintended imbalance in the concentrations of these trace elements can lead to deficiency or overload disorders as described above, which may cause some life-threatening diseases such as inflammation, arteriosclerosis, myocardial infarction, or even cancer [20].

In addition to partially obsolete methods, such as atomic absorption spectrophotometry (AAS), or flame emission spectrophotometry, modern methods, for instance inductively coupled plasma coupled with a mass spectrometry (ICP-MS) for the detection of these classes of substances, are now available since about the late 1990s. Of course, methods like AAS are still robust, high sensitive and low-cost methods for the measurement of single elements and can be used as reference methods. But the key advantages of the ICP-MS method with its speed, accuracy and simplicity preponderance the AAS.

All these methods, however, are not necessarily suitable for routine diagnostics. ICP-MS is a type of mass spectrometry that is highly sensitive enabling determination of a range of elements including trace elements. Nowadays, turnaround time plays an increasingly important role in diagnostics. The faster and more precise a deficiency of these substances is detected and corrected, the better the prognosis for the patients affected. Recently various protocols for processing biological samples have been described, e.g. a work-up of serum samples under acidic conditions using HNO_3_ [21]. For processing whole blood, however, a method in an alkaline range with ammonia or other alkaline reagents is required [22]. Here we describe a technique, allowing sequential detection of the trace elements (K, Zn, Se, Cu, Mn, Fe, Mg) in serum and whole blood by an ICP-MS method using single work-up. Because of the small amount of serum or whole blood, this method is also suitable for detecting a lack or an overload in newborns or infants.

This paper describes the development and the validation of a method for extracting a range of trace elements using 100 μl serum or whole blood only, as well as the subsequent quantification by ICP-MS.

## Materials and method

All chemicals and acids were obtained from Sigma Aldrich (Switzerland). Control samples and Calibrators were ordered from Recipe^®^ (Munich, Germany). Consumable materials like tubes, gloves and vessels were purchased from VWR (Switzerland) laboratories.

### Samples

We used commercially available control sample material, produced for quality assurance purposes (ClinCheck–Control) manufactured by Recipe^®^ Chemicals and Instruments GmbH in Munich, Germany. Therefore, no ethic approval is necessary.

### Standard and calibration

For comparative analysis of the trace element concentrations of Na, K, Zn, Se, Cu, Mn, Ca, Fe, Mg in whole blood and Cu, Se, Zn, Fe, Mg, Mn, Cr, Ni, Au and Li in serum, a serial dilution of the Recipe^®^ control samples and Recipe^®^ calibration in decreasing amounts as calibration standards had been prepared in water. Dilution and sample preparation was performed under a clean hood to prevent contamination by atmospheric particulates. Calibration standards were employed to prepare daily diluted calibration solutions. As internal standard, a mixture of Ir and Rh in a specific concentration were added to all calibration points and samples.

### Instrumentation

An Agilent 7900 ICP-MS (Agilent Technologies, Tokyo, Japan) equipped with standard nickel cones, connected to a SPS 4 Autosampler (Agilent Technologies, Tokyo, Japan) was used for all measurements. The optimization of ICP-MS was carried out using a tuning solution consisting of Cs (cesium, 55), Co (cobalt, 27), Li (lithium, 3), Mg (magnesium, 12), Tl (thallium, 81), and Y (yttrium, 39) (Agilent Technologies, Palo Alto, CA, USA). All measurements were performed in triplicates from each vial. The instrument parameters are described in Table 1. Full data were recorded with the Agilent MassHunter Data software (version 4.2).

**Table 1:** Setting parameters for the ICP-MS method.

Operating Conditions	Values
Extract 2	-250 [V]
Omega Bias	-90 [V]
Omega Lens	9,8 [V]
Deflect	-2,2 [V]
He flow	3,6 ml / min
H_2_ flow	4,8 ml / min
OctP RF	170 [V]
Forward Power	1550 W
Auxiliary gas flow	0,9 L / min
Nebulizer gas flow	1 L / min
Nebulizer type	MicroMist
Sample introduction	PeriPump
Replicates	3

Table 2 shows the acquisition parameters for the trace elements. Elements were measured in three different modes; performance check of the ICP-MS-System was performed daily.

**Table 2:** Acquisition parameters of the ICP-MS system.

		Integration m / z [sec]		
Mass	Element Name	No Gas–Mode	H_2_—Mode	He—Mode
7	Li	0,800	N/A	N/A
24	Mg	N/A	N/A	0,500
39	K	N/A	N/A	0,500
40	Ca	N/A	1,000	N/A
52	Cr	N/A	N/A	1,000
55	Mn	N/A	N/A	0,500
56	Fe	N/A	1,000	N/A
60	Ni	1,000	N/A	N/A
63	Cu	N/A	N/A	1,000
66	Zn	N/A	N/A	0,500
78	Se	N/A	1,000	N/A
103	Rh	1,000	1,000	1,000
193	Ir	1,000	1,000	1,000
197	Au	1,000	N/A	N/A

N/A = not available

### Preparation and extraction protocol

The processing protocol of the samples were optimized and modified according to the publication of Meyer et al. [23]. 100 μl of serum or whole blood ClinCheck control were added to a 15 ml polypropylene tube with screw cap (VWR laboratories). 200 μl of approximately 65% nitric acid (HNO_3_) and 100 μl of hydrogen peroxide (H_2_O_2_) were added and the mixture vortexed briefly. For dissolving, tubes then were set for 90 min at 60°C in a heating system. After incubation, samples were cooled down by adding 2100 μl ultrapure water (RT). Approximately 18 MΩ cm^-1^ ultrapure water was used for sample preparation and analysis. After vortexing and centrifugation (3 min/ 2500 rpm), samples were ready to measure.

### Method validation

The method presented was validated according to the ICH-Guidelines [24] and implemented on three different days. Validation of the method was carried out to determine performance, comprising precision in series (Cv in %), accuracy, limits of blank (LOB), limits of detection (LOD) and limits of quantification (LOQ). LOB, LOD and LOQ were calculated by quantifying blank values in three independent replicates of 3% HNO_3_ samples subsequent to sample preparation as described above. For this purpose, the corresponding amount of internal standard was added to all samples. Precision was assessed using the coefficient of variation and accuracy by analyte recovery determination.

### Particiption in an external quality assessment

External quality assessments are used to determine the performance of individual laboratories for specific measurements, and to monitor the continuing performance of laboratories. In addition, participation in an interlaboratory (external) quality program is an essential component of quality assurance and also provides evidence of laboratory competence for clinics, researchers and regulatory agencies [25]. After validating our method, we participated in an external quality assessment conducted by INSTAND e.V. (Düsseldorf Germany) for testing our validated method. The freeze-dried samples (plasma and whole blood) were transported to our laboratory at room temperature. After reconstituting the samples according to the guidelines of INSTAND e.V. the samples were ready for preparation. Descriptive statistics were calculated after standardisation of all laboratory results to percentages with reference to the true value. By subtracting 100% of these percentages, the percentage bias of the true concentration (inaccuracy) was calculated. 20% limits around the true values were considered to be appropriate threshold values for satisfactory measurements. All participating laboratories were informed about their performance within 2 months after transmitting their results.

## Results and discussion

Validation range and linearity of the method are reported in Table 3. The R^2^ value for the response function was exceeding 0,922 for all trace elements. Results of the quality control samples perfectly agree with the calculated mean values. The element composition of the serum and whole blood samples and the inter-day analysis of the samples are shown in S2 File of S1,S2 Tables. The values shown in S2 File of S1,S2 Tables are the average of triplicate preparations within a day analysis (n = 3). The final average concentration is an average concentration of the average values from each day.

**Table 3:** Linearity and validation range of serum and whole blood.

Serum		
	**R**^**2**^	**Validation Range**
Copper	0,9988	246,67–1360,00 μg/L
Selenium	0,9989	18,63–112,00 μg/L
Zinc	0,9967	350,00–1510,00 μg/L
Iron	0,9872	303,33–1480,00 μg/L
Magnesium	0,997	530,00–21500,00 μg/L
Manganese	0,9807	0,80–6,04 μg/L
Chromium	0,983	0,53–5,66 μg/L
Nickel	0,9698	0,81–5,96 μg/L
Gold	0,997	32,47–483,00 μg/L
Lithium	0,9977	1213,33–3640,00 μg/L
Whole Blood		
Sodium	0,9873	1095000,00–2210000,00 μg/L
Magnesium	0,9987	13250,00–43300,00 μg/L
Potassium	0,9673	670000,00–2180000,00 μg/L
Calcium	0,9775	23800,00–47600,00 μg/L
Manganese	0,922	7,70–22,10 μg/L
Iron	0,9811	187500,00–375000,00 μg/L
Copper	0,9981	339,00–1680,00 μg/L
Zinc	0,9865	2290,00–7980,00 μg/L
Selenium	0,9888	37,65–168,00 μg/L

Unintended imbalance in the concentrations of these trace elements can lead to deficiency or overload disorders as described above, which may cause some diseases.

Routinely, blood samples from patients are collected by puncturing a vein using a stainless steel needle, raising concerns regarding possible contaminations by such. The major components of stainless steel needles are iron, nickel, chromium and manganese. However, there is a huge variability between stainless steel needles from different suppliers [26]. Studies like Pineau et al. [27] demonstrated that these trace metals on the stainless steel needles can be reduced by rinsing them with water. Since 1995 the international Union of Pure and Applied Chemistry have been recommended using intravenous plastic needle rather than stainless steel needle when taking blood samples for analyzing trace elements [28]. Nevertheless, studies have not shown explicit verifications of significant contaminations with trace elements using stainless steel needles or plastic needles. Bro et al. [29] performed a study including 20 healthy volunteers. They conclude no differences in the content of chromium and nickel between samples taking using stainless steel needles or plastic needles. Penny and Overgaard [30] found only marginally significant differences in the contamination of chromium between samples taken using stainless steel needles and plastic needles. However, in order to exclude possible contamination, the recommendations of the international Union of Pure and Applied Chemistry should be taken into account.

Another important aspect is the impact of sample haemolysis of red blood cells in relation of some trace elements, which may have an influence on result reliability due to biological and analytical interference [31]. Some trace elements like manganese, iron or potassium show higher intracellulary concentration than extracellulary. Haemolysis of red blood cells induces the release of intracellulary trace elements. Therefore, haemolytic samples should not be analysed, or should be analysed with caution, because this can lead to falsely high values.

The processing protocol taken from Meyer et al. [23] was optimized and modified towards a more practical and more time saving method by reducing sample volume and shortening incubation time. This makes this method more suitable for routine use in biomonitoring. Compared to other studies e.g. Luna D et al. [32] we showed better results in accuracy (%) and LOD for most of the elements. In our study we only used 100 μl of serum or whole blood, which make this method also suitable for detecting trace element deficiency or excess in newborns and infants, using capillary blood sampling techniques (collection volume typically > 500 μl). [33] Basically, it should always be questioned which laboratory requirements are actually necessary, because the blood volume of a newborn is only 80–85 ml/ Kg. Several trace elements are indispensable and essential for life, others and their compounds are simply inert and others exhibit a high toxicity even in low concentrations. Mn, Fe, Cu, Zn and Se play important roles in fetal cell growth. On the other hand, overloading of these trace elements may cause toxicity to fetus and newborns [34, 35].

In this study the LOD, LOB and LOQ-values for the measurements of ^7^Li, ^24^Mg, ^39^K, ^52^Cr, ^55^Mn, ^56^Fe, ^60^ Ni, ^63^Cu ^66^Zn, ^78^Se, ^197^Au are shown in Table 4; LOD, LOB and LOQ were calculated according to Armbruster and Pry [36]. The accuracy and repeatability were calculated by analyzing replicated dilutions of the Recipe^®^ control material.

**Table 4:** LOD, LOQ and LOB of the ICP-MS method of serum and whole blood.

	Limit of blank (μg/l) (n = 9)	Limit of detection (μg/l) (n = 9)	Limit of quantification (μg/l) (n = 9)
**Serum**			
Copper	0,09	0,11	0,23
Selenium	0,15	0,19	0,33
Zinc	1,96	2,52	4,78
Iron	0,93	1,40	3,29
Magnesium	2,99	3,96	7,93
Manganese	0,11	0,15	0,30
Chromium	0,09	0,13	0,27
Nickel	0,14	0,17	0,32
Gold	0,01	0,01	0,03
Lithium	0,15	0,18	0,33
**Whole blood**			
Sodium	1206,50	1341,30	1891,40
Magnesium	477,70	759,70	1909,80
Potassium	303,00	473,00	1166,30
Calcium	83,50	127,80	308,20
Manganese	0,50	0,70	1,50
Iron	23,50	36,80	91,20
Copper	1,40	2,20	5,50
Zinc	6,50	7,10	9,50
Selenium	9,00	12,00	24,20

Linearity of the calibration curves was evaluated within the range of the Recipe^®^ calibration, control samples affording determination coefficients with R^2^ ≥0,922. Regression analysis indicated significant linear correlation between concentration and signal intensity (p < 0,05).

The method shows very good accuracy and repeatability (Tables 5 & 6) for the trace elements quantified in both, serum and whole blood.

**Table 5:** Accuracy and repeatability of the measured trace elements in serum.

Analyte	Theoretical Concentration (μg/L)	Measured Concentration (μg/L) Mean ± SD	Accuracy [%]	Repeatability C_v_ [%]
Copper	246,67	255,11 ± 4,75	103,42	1,86
	370,00	374,02 ± 1,99	101,09	0,53
	453,33	475,34 ± 9,14	104,85	1,92
	680,00	717,52 ± 22,78	105,52	3,17
	740,00	766,16 ± 22,29	103,53	2,91
	1360,00	1362,18 ± 58,52	100,16	4,30
Selenium	18,63	18,25 ± 1,12	97,96	6,14
	27,95	26,20 ± 0,72	93,73	2,75
	37,33	35,43 ± 1,24	94,90	3,50
	55,90	51,61 ± 5,09	92,33	9,86
	56,00	54,57 ± 2,77	97,44	5,08
	112,00	108,49 ± 5,72	96,87	5,27
Zinc	350,00	304,56 ± 15,69	87,02	5,15
	503,33	466,76 ± 18,19	92,73	3,90
	525,00	484,95 ± 31,35	92,37	6,46
	755,00	735,73 ± 14,86	97,45	2,02
	1050,00	1044,59 ± 27,54	99,49	2,64
	1510,00	1428,00 ± 65,52	94,57	4,59
Iron	303,33	331,81 ± 12,64	109,39	3,81
	455,00	487,69 ± 17,17	107,18	3,52
	493,33	588,55 ± 47,94	119,30	8,15
	740,00	824,84 ± 14,63	111,46	1,77
	910,00	867,97 ± 65,18	95,38	7,51
	1480,00	1479,20 ± 62,50	99,95	4,23
Magnesium	5300,00	5186,97 ± 89,10	97,87	1,72
	7166,67	7106,15 ± 127,88	99,10	1,80
	7950,00	7878,62 ± 321,30	99,16	4,08
	10750,00	10780,78 ± 343,13	100,29	3,18
	15900,00	16107,24 ± 425,26	101,30	2,64
	21500,00	20534,65 ± 753,97	95,51	3,67
Manganese	0,80	0,83 ± 0,06	103,90	7,23
	1,21	1,20 ± 0,14	99,98	11,67
	2,01	2,49 ± 0,55	123,69	22,09
	2,41	2,10 ± 0,35	87,01	16,67
	3,02	3,31 ± 0,40	109,72	12,08
	6,04	5,85 ± 0,38	96,94	6,50
Chromium	0,59	1,28 ± 0,17	214,98	13,28
	0,89	1,38 ± 0,16	155,15	11,59
	1,78	2,10 ± 0,26	117,99	12,38
	1,89	3,75 ± 1,88	118,45	50,13
	2,83	4,53 ± 1,12	124,76	24,72
	5,66	5,65 ± 0,30	99,76	5,31
Analyte	Theoretical concentrations	Measured Concentration (μg/L) Mean ± SD	Accuracy [%]	Repeatability C_v_ [%]
Nickel	0,81	1,05 ± 0,26	129,49	24.76
	1,22	1,90 ± 0,18	156,53	9.47
	1,99	2,14 ± 0,27	107,87	12.62
	2,43	3,33 ± 0,16	136,94	4.80
	2,98	3,34 ± 0,34	111,93	10.18
	5,96	6,00 ± 0,64	100,64	10.67
Gold	32,47	38,97 ± 5,17	120,02	13.27
	48,70	64,29 ± 8,29	132,02	12.89
	97,40	101,57 ± 2,24	104,28	2.21
	161,00	171,99 ± 13,59	106,83	7.90
	241,50	267,24 ± 21,33	110,66	7.98
	483,00	512,87 ± 54,24	106,19	10.58
Lithium	1213,33	1161,43 ± 26,59	95,72	2.29
	1820,00	1736,70 ± 34,91	95,42	2.01
	2406,67	2194,99 ± 17,14	91,20	0.78
	3610,00	3437,43 ± 89,67	95,22	2.61
	3640,00	3678,20 ± 120,63	101,05	3.28
	7220,00	6889,48 ± 265,25	95,42	3.85

**Table 6:** Accuracy and repeatability of the measured trace elements in whole blood.

Analyte	Theoretical Concentration (μg/L)	Measured Concentration (μg/L) Mean ± SD	Accuracy [%]	Repeatability C_v_ [%]
Sodium	1095000	938069,75 ± 22445,18	85,67	2,39
	1095000	963072,28 ± 31214,29	87,95	3,24
	1105000	1073121,30 ± 2964,07	97,12	0,28
	2190000	1889717,80 ± 47427,50	86,29	2,51
	2190000	2005786,19 ± 29856,53	91,59	1,49
	2210000	2034499,42 ± 64855,44	92,06	3,19
Magnesium	13250	12897,04 ± 628,02	97,34	4,87
	17450	16734,75 ± 789,75	95,90	4,72
	21650	21105,16 ± 382,05	97,48	1,81
	26500	24478,03 ± 543,44	92,37	2,22
	34900	32592,08 ± 463,56	93,39	1,42
	43300	39513,01 ± 518,39	91,25	1,31
Potassium	670000	634366,81 ± 21801,69	94,68	3,44
	880000	833121,09 ± 23972,65	94,67	2,88
	1090000	986309,60 ± 19680,13	90,49	2,00
	1340000	1172996,44 ± 52933,82	87,54	4,51
	1760000	1511923,57 ± 75412,86	85,90	4,99
	2180000	2223448,50 ± 106555,26	101,99	4,79
Calcium	23800	19600,41 ± 599,36	82,35	3,06
	23800	21608,76 ± 1304,46	90,79	6,04
	23850	21477,18 ± 729,15	90,05	3,39
	47600	44225,27 ± 2226,55	92,91	5,03
	47600	43826,45 ± 609,06	92,07	1,39
	47700	49460,14 ± 393,61	103,69	0,80
Manganese	4,44	5,01 ± 1,24	112,92	24,75
	7,70	7,46 ± 1,11	96,86	14,88
	8,87	8,38 ± 1,81	94,48	21,60
	11,05	10,20 ± 1,69	92,35	16,57
	15,40	12,55 ± 0,26	81,50	2,07
	22,10	26,54 ± 1,56	120,10	5,88
Iron	187500	164985,76 ± 11946,26	87,99	7,24
	187500	171830,79 ± 9332,22	91,64	5,43
	187500	183436,39 ± 4125,26	97,83	2,25
	375000	297550,28 ± 5688,77	79,35	1,91
	375000	310154,58 ± 4684,50	82,71	1,51
	375000	324810,47 ± 17702,80	86,62	5,45
Copper	339,00	311,05 ± 77,16	91,76	24,81
	555,00	501,58 ± 93,30	90,37	18,60
	678,00	650,26 ± 27,64	95,91	4,25
	840,00	748,40 ± 32,02	89,09	4,28
	1110,00	1025,60 ± 67,85	92,40	6,62
	1680,00	1505,50 ± 61,76	89,61	4,10
Zinc	2290,00	2138,61 ± 64,69	93,39	3,02
	3135,00	3013,66 ± 219,78	96,13	7,29
	3990,00	3630,23 ± 145,68	90,98	4,01
	4580,00	4328,68 ± 368,84	94,51	8,52
	6270,00	6119,93 ± 132,87	97,61	2,17
	7980,00	6995,66 ± 50,89	87,66	0,73
Selenium	37,65	50,48 ± 4,73	134,08	9,37
	75,30	84,07 ± 12,03	111,64	14,31
	84,00	93,43 ± 2,97	111,23	3,18
	84,00	82,86 ± 4,97	98,64	6,00
	168,00	163,17 ± 2,62	97,13	1,61
	168,00	174,01 ± 12,58	103,58	7,23

**Table 7:** Comparison of the reference ranges established in this work with values in the literature.

Analyte in serum	validation range in this study	Reference ranges in literature
Copper	246,67–1360,00 μg/L	600,00–1400,00 μg/L [37]
Selenium	18,63–112,00 μg/L	30,00–105,00 μg/L [37]
Zinc	350,00–1510,00 μg/L	600,00–1200,00 μg/L [37]
Iron	303,33–1480,00 μg/L	1100,00–1300,00 μg/L [37]
Magnesium	530,00–21500,00 μg/L	16044,60–26011,70 μg/L [38]
Manganese	0,80–6,04 μg/L	0,10–2,90 μg/L [37]
Chromium	0,53–5,66 μg/L	< 0,40 μg/L [38]
Nickel	0,81–5,96 μg/L	< 2,00 μg/L [38]
Gold	32,47–483,00 μg/L	N/A
Lithium	1213,33–3640,00 μg/L	4164,00–5205,00 μg/L [39]
Analyte in whole blood	validation range in this study	Reference ranges in literature
Sodium	1095000,00–2210000,00 μg/L	N/A
Magnesium	13250,00–43300,00 μg/L	30000,00–40000,00 μg/L [38]
Potassium	670000,00–2180000,00 μg/L	N/A
Calcium	23800,00–47600,00 μg/L	N/A
Manganese	7,70–22,10 μg/L	8,30–15,00 μg/L [38]
Iron	187500,00–375000,00 μg/L	425000,00–500000,00 μg/L [40]
Copper	339,00–1680,00 μg/L	700,00–1390,00 μg/L [38]
Zinc	2290,00–7980,00 μg/L	4500,00–7500.00 μg/L [38]
Selenium	37,65–168,00 μg/L	90,00–230,00 μg/L [38]

N/A = not available

The method presented was assessed by analyzing aliquots and dilutions of human blood material with known concentrations of several elements (ClinCheck-Control, serum and whole blood, RECIPE Chemicals + Instruments GmbH, Munich, Germany); all samples were analyzed in triplicates. The selected validation areas for each element were based on the physiological areas published in literature (Table 7). With our described method and our chosen validation points, we cover the physiological areas described in the literature for most of the elements.

Effective reduction of sample matrix extraction and optimization procedures made it possible to avoid most of the interference problems and matrix-induced ionization effects in analysis. Accuracy was found to be between 80–130%, for all elements analysed (except for Selenium in whole blood, Chromium, Nickel and Gold in serum at lower concentrations) in both, serum and whole blood. In fact, the range is in agreement with data presented in the study of Burrows et al. [41] while trace element determination in serum shows slightly better accuracies for copper, zinc, selenium and magnesium compared to whole blood samples which may be due to less matrix effects in serum samples. Whole blood contains high concentrations of inorganic ions. It also contains a numerous of diverse organic compounds such as lipids, carbohydrates and proteins of a wide range of molecular weights. These components can cause analytical interferences when the matrix is not completely decomposed. Repeatabilities (C_v_) of all elements were below 15% except for manganese, nickel and copper at a low concentration in whole blood and serum samples as being close to the LOQ; calculated concentrations, however, were in agreement with certified values for all elements.

Validation ranges of elements (Table 3) were linear across concentrations measured for each element with R^2^ values exceeding 0,922. Ir and Rh were used as internal standards to eliminate or reduce variations such as power fluctuations, temperature changes, differences in solution parameters (e.g. density, viscosity, or surface tension) and changes in the sample introduction system [42,43].

After validating the method, we participated in an external quality assessment for most of the validated elements, conducted by INSTAND e.V.. The results are shown in Table 8.

**Table 8:** Results of the external quality assessment.

**Analyte in serum**	**Unit**	**Sample Nr.**	**Measured value**	**Target value**	**deviation**
Copper	μg/L	21	1550,62	1652,30	-6,20%
		22	724,47	775,31	-5,80%
Selenium	μg/L	21	153,00	137,00	11,80%
		22	161,00	156,00	3,30%
Zinc	μg/L	21	2460	2560	-3,90%
		22	1970	2070	-4,80%
Iron	μg/L	21	955,04	977,38	-2,30%
		22	1541,46	1731,35	-11,00%
Manganese	μg/L	21	4,20	5,63	-25,40%
		22	9,60	10,70	-10,30%
**Analyte in whole blood**	**Unit**	**Sample Nr.**	**Measured value**	**Target value**	**deviation**
Magnesium	μg/L	61	36200,00	37200,00	-2,70%
		62	40200,00	41900,00	-4,10%
Manganese	μg/L	61	14,10	13,60	3,30%
		62	17,40	16,10	8,30%
Selenium	μg/L	61	127,00	123,00	3,00%
		62	150,00	146,00	2,50%
Zinc	μg/L	61	4910,00	4970,00	-1,20%
		62	54600,00	46500,00	17,40%

If the trace element analysis are used to check a lack or an overload in the human body, or are used to determine the compliance with the regulations, any error in the result can have extremely serious consequences. For an independent laboratory, producing unreliable test results taints its reputation and the trust of its customers. It may lose them for ever. After measuring and transmitting the values, INSTAND e.V. issued us with a certificate of good standard for each trace element we recorded.

Our data and the subsequent participation in an external quality assessment demonstrate, that the method is valid for quantification of the respective elements in both, human serum and whole blood, requiring only one-step preparation with 100 μl of sample; it is cost-effective and fast without noteworthy matrix effects for all trace elements measured and is also suitable for the measurement of newborns and infants because of its less sample volume.

## Conclusions

Serum and whole blood are the two most widely used matrices in clinical laboratories. During the last years, interest in the role of trace elements in biological systems has increased. Unintended imbalance in the concentrations of these trace elements can lead to deficiency or overload disorders as described above. The described method allows the sequential detection of the trace elements (K, Zn, Se, Cu, Mn, Fe, Mg) in serum and whole blood by an ICP-MS method using a single work-up out of 100 μl only. Results show that trace element concentrations measured with the validated ICP-MS method can be valuable to research and routine diagnostics with the method offering excellent, rapid, precise, inexpensive and timely quantitation in low-concentration multielement analysis.

## Supporting information

S1 File Graphical abstract. (TIF)

S2 File (DOCX)
