# Supplementary figures and images for: ICP-MS trace element analysis in serum and whole blood
Source: PLoS One. 2020 May 20;15(5):e0233357. doi: 10.1371/journal.pone.0233357 (PMC7239469; doi:10.1371/journal.pone.0233357)

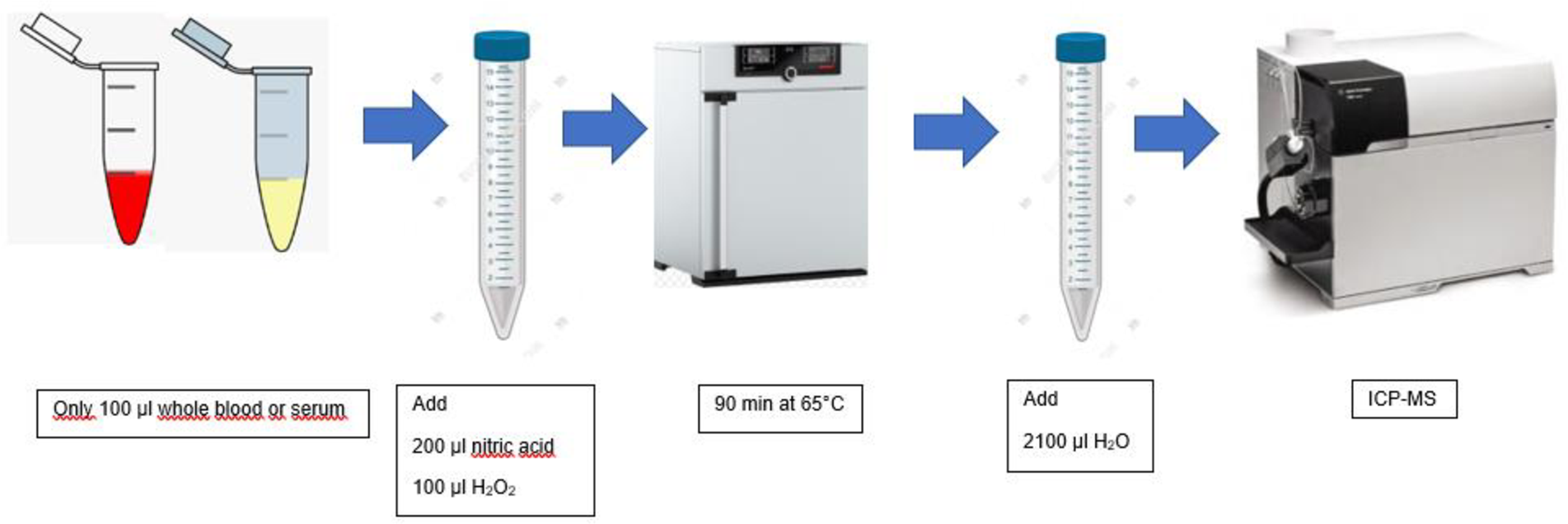

Supplement: S1 File — (TIF) [file pone.0233357.s001.tif]
