# Supplementary material for: ICP-MS trace element analysis in serum and whole blood
Source: PLoS One. 2020 May 20;15(5):e0233357. doi: 10.1371/journal.pone.0233357 (PMC7239469; doi:10.1371/journal.pone.0233357)
Supplement: S2 File — (DOCX) [file pone.0233357.s002.docx]

**Supporting information**


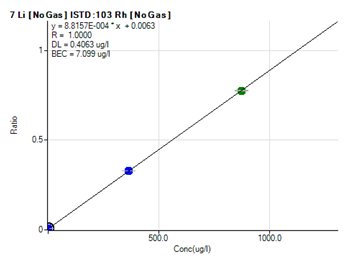

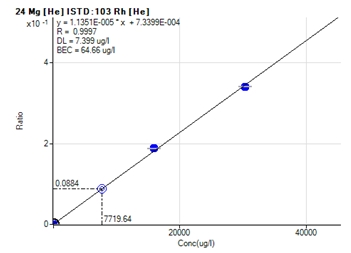


Calibration curve for Li Calibration curve for Mg


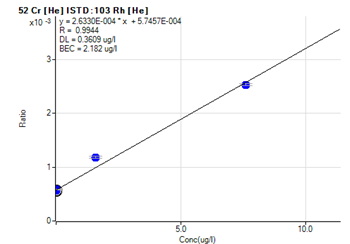

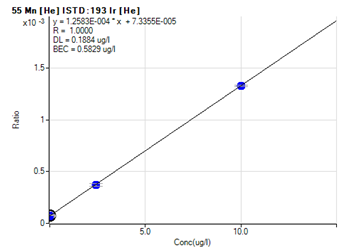


Calibration curve for Cr Calibration curve for Mn


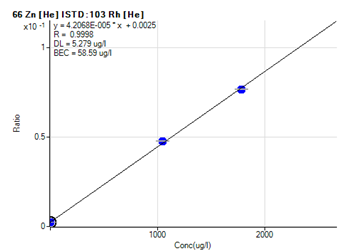

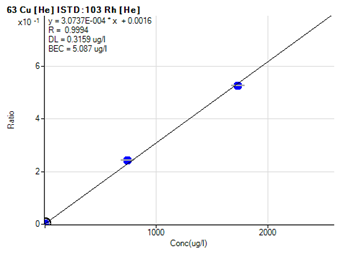


Calibration curve for Zn Calibration curve for Cu


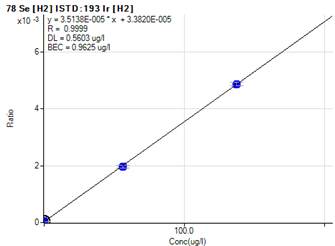

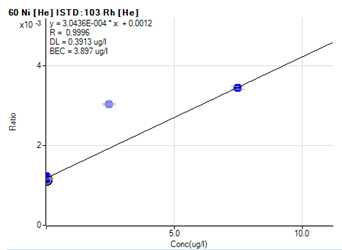


Calibration curve for Se Calibration curve for Ni


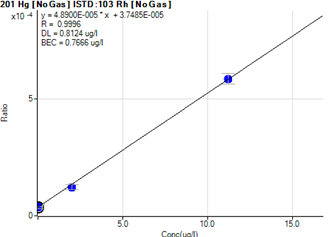


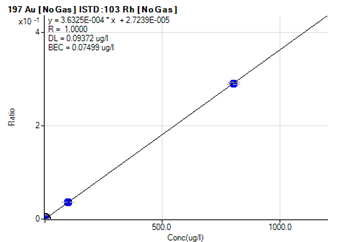


Calibration curve for Au Calibration curve for Hg

**Figure 1: Typical calibration curves obtained with the method**

**Table S1 Serum interday analysis results (µg/L each element)**

| **Sample** | **Average** | **Li** | **Mg** | **Cr** | **Mn** | **Fe** | **Ni** | **Cu** | **Zn** | **Se** | **Au** |
| --- | --- | --- | --- | --- | --- | --- | --- | --- | --- | --- | --- |
| 1 | Day 1 | 3833,41 | 16296,23 | 1,74 | 1,61 | 908,59 | 3,54 | 774,77 | 1047,40 | 55,68 | 98,81 |
|  | Day 2 | 3661,92 | 16507,20 | 2,30 | 2,39 | 919,32 | 3,32 | 788,11 | 1076,83 | 54,72 | 101,59 |
|  | Day 3 | 3539,27 | 15518,30 | 2,26 | 2,29 | 776,00 | 3,13 | 735,58 | 1009,55 | 44,44 | 104,30 |
|  | MW | 3678,20 | 16107,24 | 2,10 | 2,10 | 867,97 | 3,33 | 766,16 | 1044,59 | 51,61 | 101,57 |
|  | Cv (%) | 3,28 | 2,64 | 12,15 | 16,51 | 7,51 | 4,95 | 2,91 | 2,64 | 9,86 | 2,21 |
|  | Std | 12,06 | 425,26 | 0,26 | 0,35 | 65,18 | 0,16 | 22,29 | 27,54 | 5,09 | 2,24 |
|  | RSD | 0,03 | 0,03 | 0,12 | 0,17 | 0,08 | 0,05 | 0,03 | 0,03 | 0,10 | 0,02 |
| 2 | Day 1 | 1708,13 | 7589,48 | 1,23 | 1,00 | 481,03 | 1,66 | 363,45 | 447,93 | 27,11 | 74,82 |
|  | Day 2 | 1716,12 | 7719,64 | 1,30 | 1,31 | 470,79 | 2,08 | 367,83 | 482,34 | 25,34 | 54,57 |
|  | Day 3 | 1785,86 | 8326,75 | 1,61 | 1,30 | 511,25 | 1,97 | 390,78 | 524,58 | 26,14 | 63,49 |
|  | MW | 1736,70 | 7878,62 | 1,38 | 1,20 | 487,69 | 1,90 | 374,02 | 484,95 | 26,20 | 64,29 |
|  | Cv (%) | 2,01 | 4,08 | 11,72 | 12,02 | 3,52 | 9,51 | 3,21 | 6,46 | 2,77 | 12,89 |
|  | Std | 3,49 | 321,30 | 0,16 | 0,14 | 17,17 | 0,18 | 11,99 | 31,35 | 0,72 | 8,29 |
|  | RSD | 0,02 | 0,04 | 0,12 | 0,12 | 0,04 | 0,10 | 0,03 | 0,06 | 0,03 | 0,13 |
| 3 | Day 1 | 1187,01 | 5274,21 | ND | ND | 340,31 | 1,03 | 252,94 | 283,52 | 19,53 | 45,76 |
|  | Day 2 | 1124,77 | 5064,61 | 1,44 | 0,90 | 313,95 | 1,38 | 261,71 | 308,99 | 16,81 | 33,21 |
|  | Day 3 | 1172,52 | 5222,08 | 1,11 | 0,77 | 341,17 | 0,74 | 250,69 | 321,18 | 18,42 | 37,93 |
|  | MW | 1161,43 | 5186,97 | 1,28 | 0,83 | 331,81 | 1,05 | 255,11 | 304,56 | 18,25 | 38,97 |
|  | Cv (%) | 2,29 | 1,72 | 13,24 | 7,33 | 3,81 | 24,74 | 1,86 | 5,15 | 6,12 | 13,27 |
|  | Std | 2,66 | 89,10 | 0,17 | 0,06 | 12,64 | 0,26 | 4,75 | 15,69 | 1,12 | 5,17 |
|  | RSD | 0,02 | 0,02 | 0,13 | 0,07 | 0,04 | 0,25 | 0,02 | 0,05 | 0,06 | 0,13 |
| 4 | Day 1 | 7075,16 | 21125,47 | 5,46 | 6,18 | 1560,30 | 5,10 | 1421,36 | 1491,62 | 115,90 | 582,62 |
|  | Day 2 | 7078,92 | 21007,95 | 6,07 | 6,07 | 1469,10 | 6,53 | 1382,69 | 1454,52 | 107,61 | 505,68 |
|  | Day 3 | 6514,37 | 19470,53 | 5,42 | 5,32 | 1408,21 | 6,36 | 1282,49 | 1337,85 | 101,98 | 450,33 |
|  | MW | 6889,48 | 20534,65 | 5,65 | 5,85 | 1479,20 | 6,00 | 1362,18 | 1428,00 | 108,49 | 512,87 |
|  | Cv (%) | 3,85 | 3,67 | 5,26 | 6,49 | 4,23 | 10,63 | 4,30 | 4,59 | 5,27 | 10,58 |
|  | Std | 26,52 | 753,97 | 0,30 | 0,38 | 62,50 | 0,64 | 58,52 | 65,52 | 5,72 | 54,24 |
|  | RSD | 0,04 | 0,04 | 0,05 | 0,06 | 0,04 | 0,11 | 0,04 | 0,05 | 0,05 | 0,11 |
| 5 | Day 1 | 3475,39 | 10842,83 | 4,01 | 2,77 | 843,99 | 3,11 | 730,74 | 740,28 | 57,19 | 292,43 |
|  | Day 2 | 3313,66 | 10332,95 | 6,08 | 3,72 | 808,47 | 3,82 | 685,47 | 715,68 | 50,73 | 240,28 |
|  | Day 3 | 3523,24 | 11166,56 | 3,50 | 3,45 | 822,05 | 3,08 | 736,35 | 751,22 | 55,77 | 269,02 |
|  | MW | 3437,43 | 10780,78 | 4,53 | 3,31 | 824,84 | 3,34 | 717,52 | 735,73 | 54,57 | 267,24 |
|  | Cv (%) | 2,61 | 3,18 | 24,61 | 12,10 | 1,77 | 10,23 | 3,17 | 2,02 | 5,08 | 7,98 |
|  | Std | 8,97 | 343,13 | 1,12 | 0,40 | 14,63 | 0,34 | 22,78 | 14,86 | 2,77 | 21,33 |
|  | RSD | 0,03 | 0,03 | 0,25 | 0,12 | 0,02 | 0,10 | 0,03 | 0,02 | 0,05 | 0,08 |
| 6 | Day 1 | 2215,33 | 7026,16 | 2,00 | 1,95 | 543,31 | 2,51 | 474,87 | 444,20 | 36,80 | 187,34 |
|  | Day 2 | 2196,22 | 7005,69 | 2,49 | 3,25 | 654,90 | 2,03 | 464,39 | 467,25 | 33,80 | 154,30 |
|  | Day 3 | 2173,40 | 7286,62 | 6,21 | 2,27 | 567,44 | 1,89 | 486,76 | 488,75 | 35,69 | 174,34 |
|  | MW | 2194,99 | 7106,15 | 3,57 | 2,49 | 588,55 | 2,14 | 475,34 | 466,73 | 35,43 | 171,99 |
|  | Cv (%) | 0,78 | 1,80 | 52,74 | 22,18 | 8,15 | 12,43 | 1,92 | 3,90 | 3,49 | 7,90 |
|  | Std | 1,71 | 127,88 | 1,88 | 0,55 | 47,94 | 0,27 | 9,14 | 18,19 | 1,24 | 13,59 |
|  | RSD | 0,01 | 0,02 | 0,53 | 0,22 | 0,08 | 0,12 | 0,02 | 0,04 | 0,03 | 0,08 |

ND = not detected. Std = Standard deviation

**Table S2 Whole blood interday analysis results (µg/L each element)**

| **Sample** | **Average** | **Na** | **Mg** | **K** | **Ca** | **Mn** | **Fe** | **Cu** | **Zn** | **Se** |
| --- | --- | --- | --- | --- | --- | --- | --- | --- | --- | --- |
| 1 | Day 1 | 1825556,75 | 25240,57 | 1237106,94 | 43301,49 | 8,81 | 306539,84 | 611,91 | 4110,71 | 81,87 |
|  | Day 2 | 1938726,74 | 24179,77 | 1107469,31 | 47294,13 | 10,35 | 319122,77 | 662,85 | 4027,27 | 99,78 |
|  | Day 3 | 1904869,92 | 24013,74 | 1174413,08 | 42080,18 | 5,98 | 348768,80 | 676,01 | 4848,07 | 70,56 |
|  | MW | 1889717,80 | 24478,03 | 1172996,44 | 44225,27 | 8,38 | 324810,47 | 650,26 | 4328,68 | 84,07 |
|  | Cv % | 2,51 | 2,22 | 4,51 | 5,03 | 21,59 | 5,45 | 4,25 | 8,52 | 14,31 |
|  | Std | 47427,50 | 543,44 | 52933,82 | 2226,55 | 1,81 | 17702,80 | 27,64 | 368,84 | 12,03 |
|  | RSD | 0,03 | 0,02 | 0,05 | 0,05 | 0,22 | 0,05 | 0,04 | 0,09 | 0,14 |
| 2 | Day 1 | 909222,17 | 12371,68 | 612276,66 | 19112,27 | 5,01 | 171468,18 | 404,80 | 2190,39 | 55,34 |
|  | Day 2 | 963962,72 | 13779,89 | 626784,23 | 20444,59 | 6,53 | 160586,82 | 312,55 | 2178,03 | 52,03 |
|  | Day 3 | 941024,36 | 12539,54 | 664039,55 | 19244,37 | 3,48 | 183437,37 | 215,80 | 2047,41 | 44,07 |
|  | MW | 938069,75 | 12897,04 | 634366,81 | 19600,41 | 5,01 | 171830,79 | 311,05 | 2138,61 | 50,48 |
|  | Cv % | 2,39 | 4,87 | 3,44 | 3,06 | 24,84 | 5,43 | 24,81 | 3,02 | 9,37 |
|  | Std | 22445,18 | 628,02 | 21801,69 | 599,36 | 1,24 | 9332,22 | 77,16 | 64,69 | 4,73 |
|  | RSD | 0,02 | 0,05 | 0,03 | 0,03 | 0,25 | 0,05 | 0,25 | 0,03 | 0,09 |
| 3 | Day 1 | 1944008,53 | 33237,27 | 1562778,00 | 49366,97 | 12,81 | 304464,67 | 1052,90 | 5957,97 | 191,72 |
|  | Day 2 | 2066787,56 | 32370,15 | 1405311,27 | 49982,02 | 12,19 | 297654,91 | 932,28 | 6283,43 | 163,75 |
|  | Day 3 | 2092702,16 | 32168,81 | 1567681,45 | 49031,44 | 12,65 | 290531,27 | 1091,62 | 6118,37 | 166,55 |
|  | MW | 2034499,42 | 32592,08 | 1511923,57 | 49460,14 | 12,55 | 297550,28 | 1025,60 | 6119,93 | 174,01 |
|  | Cv % | 3,19 | 1,42 | 4,99 | 0,80 | 2,11 | 1,91 | 6,62 | 2,17 | 7,23 |
|  | Std | 64855,44 | 463,56 | 75412,86 | 393,62 | 0,26 | 5688,77 | 67,85 | 132,87 | 12,58 |
|  | RSD | 0,03 | 0,01 | 0,05 | 0,01 | 0,02 | 0,02 | 0,07 | 0,02 | 0,07 |
| 4 | Day 1 | 1074127,13 | 17836,15 | 866395,02 | 22183,81 | 8,89 | 181372,98 | 631,31 | 3291,12 | 92,30 |
|  | Day 2 | 1076142,56 | 16344,58 | 810856,86 | 21774,26 | 7,32 | 189193,92 | 457,59 | 2753,61 | 97,50 |
|  | Day 3 | 1069094,22 | 16023,54 | 822111,38 | 20473,48 | 6,17 | 179742,26 | 415,84 | 2996,24 | 90,50 |
|  | MW | 1073121,30 | 16734,75 | 833121,09 | 21477,18 | 7,46 | 183436,39 | 501,58 | 3013,66 | 93,43 |
|  | Cv % | 0,28 | 4,72 | 2,88 | 3,40 | 14,95 | 2,25 | 18,60 | 7,29 | 3,18 |
|  | Std | 2964,07 | 789,75 | 23972,65 | 729,15 | 1,11 | 4125,26 | 93,30 | 219,78 | 2,97 |
|  | RSD | 0,00 | 0,05 | 0,03 | 0,03 | 0,15 | 0,02 | 0,19 | 0,07 | 0,03 |
| 5 | Day 1 | 2034471,43 | 39154,32 | 2289497,12 | 44034,47 | 27,80 | 312583,13 | 1452,38 | 6935,88 | 166,80 |
|  | Day 2 | 1964611,02 | 39138,64 | 2073124,49 | 42998,58 | 24,35 | 314278,23 | 1472,01 | 6990,85 | 162,00 |
|  | Day 3 | 2018276,13 | 40246,06 | 2307723,88 | 44446,30 | 27,48 | 303602,38 | 1592,11 | 7060,25 | 160,73 |
|  | MW | 2005786,19 | 39513,01 | 2223448,50 | 43826,45 | 26,54 | 310154,58 | 1505,50 | 6995,66 | 163,17 |
|  | Cv % | 1,49 | 1,31 | 4,79 | 1,39 | 5,87 | 1,51 | 4,10 | 0,73 | 1,60 |
|  | Std | 29856,53 | 518,39 | 106555,26 | 609,06 | 1,56 | 4684,50 | 61,76 | 50,89 | 2,62 |
|  | RSD | 0,01 | 0,01 | 0,05 | 0,01 | 0,06 | 0,02 | 0,04 | 0,01 | 0,02 |
| 6 | Day 1 | 936967,71 | 20942,51 | 971338,19 | 19791,24 | 12,60 | 179860,03 | 790,11 | 3801,45 | 84,57 |
|  | Day 2 | 1006953,31 | 20740,27 | 973476,50 | 22243,87 | 9,14 | 164486,68 | 712,27 | 3445,39 | 76,10 |
|  | Day 3 | 945295,82 | 21632,69 | 1014114,10 | 22791,18 | 8,88 | 150610,56 | 742,81 | 3643,84 | 87,91 |
|  | MW | 963072,28 | 21105,16 | 986309,60 | 21608,76 | 10,20 | 164985,76 | 748,40 | 3630,23 | 82,86 |
|  | Cv % | 3,24 | 1,81 | 2,00 | 6,04 | 16,60 | 7,24 | 4,28 | 4,01 | 6,00 |
|  | Std | 31214,29 | 382,05 | 19680,13 | 1304,46 | 1,69 | 11946,26 | 32,02 | 145,68 | 4,97 |
|  | RSD | 0,03 | 0,02 | 0,02 | 0,06 | 0,17 | 0,07 | 0,04 | 0,04 | 0,06 |

Std = Standard deviation
